# Discharging Preterm Infants on Caffeine—Practise Variation Across Europe: Results of a Cross‐Sectional Survey

**DOI:** 10.1111/apa.70502

**Published:** 2026-03-14

**Authors:** Martin Kuntz, Lina Raljevic, Katharina Bergmann, Daniel Matheisl, Hendryk Schneider, Hans Fuchs

**Affiliations:** ^1^ Department of General Pediatrics, Adolescent Medicine and Neonatology, Medical Center and Faculty of Medicine University of Freiburg Germany

**Keywords:** apnea of prematurity, caffeine, discharge planning, survey

## Abstract

**Aim:**

Caffeine is the mainstay of treatment for apnea of prematurity, yet the optimal strategy and timing for discontinuation remain undefined. Marked inter‐centre variation in the practise of discharging preterm infants home on caffeine therapy has been reported. We aimed to map current practises in Europe concerning discharge of preterm infants on caffeine therapy, including home‐monitoring and strategies for discontinuing ambulatory therapy.

**Methods:**

A web‐based three‐part questionnaire was sent to 633 neonatal units in 36 European countries (July–October 2025). One response per unit was permitted. The survey collected data on policies and practises regarding discharge on caffeine, criteria for stopping therapy, and post‐discharge monitoring. Responses were analysed descriptively.

**Results:**

Complete responses were obtained from 125 units in 21, revealing wide variation in practise patterns. Fifty‐eight per cent of participating centres reported discharging preterm infants on continued caffeine therapy at least occasionally. Among those units, 58% reported prescribing home‐monitoring routinely for infants discharged on caffeine therapy. Overall, significant differences in practise existed between geographical regions but not between high‐ and low‐volume centres.

**Conclusions:**

Discharging preterm infants on continued caffeine therapy is a widespread practise among neonatologists in Europe, with considerable variation among regions and differing strategies concerning home‐monitoring and discontinuation of treatment.

## Summary

Existing data suggest that discharging preterm infants on continued caffeine therapy is performed at least in some countries, but evidence and guidelines on this matter are lacking. Of 125 neonatal units across 21 European countries, 58% of centres prescribe caffeine therapy for preterm infants after discharge at least occasionally. There is wide variation in required monitoring and timing of cessation with marked geographic differences.

**Abbreviations**
mg/kg/d: milligrams per kilogram bodyweight per day
NICU: neonatal intensive care unit
PMA: post‐menstrual age
VLBW: very low birth weight

## 1 Introduction

Following the results of the CAP (Caffeine for Apnea of Prematurity) trial [1], caffeine therapy has become standard of care for very low birth weight (VLBW) infants. While its benefits on apnea of prematurity, bronchopulmonary dysplasia, and on long‐term neurocognitive outcomes [2] are clear, questions remain open concerning timing, dosing, duration, and cessation of therapy. Higher doses and longer courses of caffeine therapy have been studied mostly with promising results [3, 4, 5, 6], with the exception of very high doses [7]. Furthermore, there is still an ongoing debate concerning the optimal time point for discontinuation of caffeine therapy in preterm infants. There is controversy over whether preterm infants with persisting apnea benefit from prolonged therapy or even continuation of therapy after discharge. The role of ambulatory caffeine therapy in preterm infants has not been systematically studied and is mostly governed by local protocols or individual practise. Most national and international guidelines do not address this issue [8]. Data from France, the United States, and Canada suggest considerable variation in practise [9, 10, 11]. However, for most European countries, such data is lacking. To inform future studies and guideline development, we aimed to collect systematic data on current practise concerning discharge planning and home therapy with caffeine in preterm infants across Europe.

## 2 Methods

We conducted a web‐based, three‐part survey about several aspects of respiratory management of preterm infants among European neonatologists. Here, we report on the part concerning discharge on caffeine therapy. The other parts will be reported separately.

Questions and answer options were developed and refined through several iterative rounds by the authors. The draft was pilot‐tested by members of our unit not involved in the study to correct technical errors and ambiguous wording.

As many neonatal units in European countries as possible were identified, building on the list we used in a prior survey [12]. In addition, we used an online search and public directories to identify possible participants. A total of 633 units in 36 countries were identified. An e‐mail, containing a link to the online survey, was sent to the head of each department, explaining the study, assuring confidentiality, and requesting participation. It was explained that the survey could be completed by another medical team member, but that only one answer from each department was allowed. The survey was open from July to October 2025. Two reminder e‐mails were sent to possible participants in order to increase response rate.

The survey was conducted on a REDCap [13] instance hosted by the University Medical Center Freiburg. Survey links were intentionally not personalised to minimise collection of personal data.

Questions asked in this survey centred around practises concerning discharge of preterm infants on caffeine therapy and included questions on dosing, criteria for discontinuation, and home monitoring. In addition, we asked for the country in which the unit is located as well as data on volume. The full survey can be found in Appendix S1.

Statistical analyses were performed using R v4.4.1 [14]. For all comparisons, Fisher's exact test with Monte Carlo simulation (10^6^ replicates) was used with a set random seed for reproducibility.

## 3 Results

From the 633 units contacted in 36 European countries, we received 157 responses, of which 125 from 21 countries were complete, translating to a response rate of 20%. Details can be found in Table S1.

Eighty‐two per cent of respondents identified their institution as a level III/IV centre (as defined by the American Academy of Pediatrics [15]). Fifty‐seven per cent of participants reported treating more than 50 VLBW infants per year in their respective institution, which we defined as ‘high‐volume’ for the purpose of this report.

### 3.1 Discontinuation of Caffeine Therapy

Most centres (73%) use a combination of respiratory stability and post‐menstrual age (PMA) to decide when to discontinue caffeine therapy in preterm infants. Twenty‐six per cent rely solely on PMA, and only 2% discontinue caffeine as soon as the infant is off ventilation.

In stable infants, most centres discontinue caffeine between 34 + 0 and 36 + 6 weeks PMA (74%) or between 32 + 0 and 33 + 6 weeks PMA (22%). Very few centres discontinue caffeine earlier (< 32 weeks PMA) or later (≥ 37 weeks PMA) on a regular basis.

**FIGURE 1:**
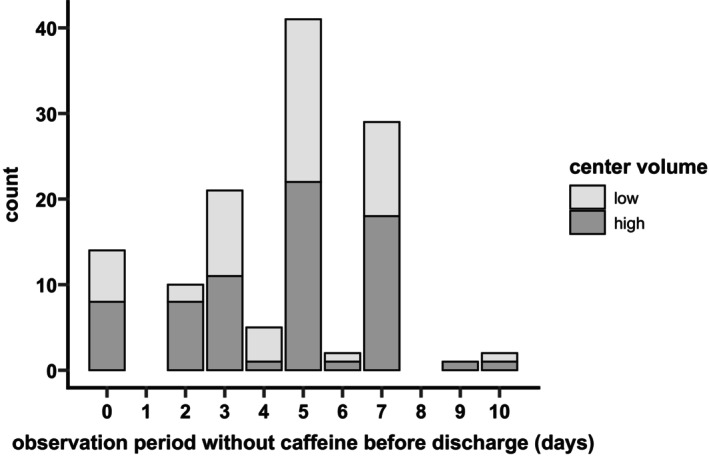
Count of centres reporting specific observation periods after discontinuation of caffeine therapy before discharge. Centre volume is indicated.

After discontinuing caffeine, the majority of centres (89%) report an observational period of several days before discharge. Concerning the number of days off caffeine required before discharge, there is wide variation in practise. Three, five and seven days were the most frequently reported intervals. There was no apparent difference between high‐ and low‐volume units. Details can be found in Figure 1.

Fifty‐eight per cent of participating units reported discharging preterm infants on caffeine therapy at least on an individual basis.

There was significant variability in respondents' practises related to home monitoring, dosing and caffeine cessation after discharge.

Most centres reported a standard dose of caffeine of > 5 and < 10 mg/kg/d (41%), followed by 5 mg/kg/d (38%) and 10 mg/kg/d (18%). Higher doses were reported rarely. Once‐daily dosing is prescribed most commonly (70%), and most clinicians do not adjust the dose as the infants grow after discharge (72%).

The majority of respondents prescribe home monitoring as a standard precaution in preterm infants discharged on caffeine; 58% do so routinely and 19% base the decision on individual factors.

Most centres schedule routine outpatient visits of preterm infants discharged on caffeine therapy. Monthly (35%) and less‐than‐monthly (28%) intervals were reported most frequently. Some centres (8%) schedule outpatients visits for these infants more often, namely every 2 weeks. A relevant proportion of centres (29%) reported no routine outpatient visits. Interestingly, even in centres stating to prescribe home monitoring routinely in infants discharged on caffeine, 28% do not schedule routine outpatient visits for these patients.

Concerning the decision to discontinue caffeine therapy after discharge, most centres (82%) rely on individualised criteria. Eighteen percent reported cessation of caffeine therapy at a fixed timepoint, either at a certain timepoint after discharge (3–12 weeks) or at a specific PMA (4–8 weeks after due date).

For individual criteria, a combination of monitor readings, history and current wellbeing was named most often. Two centres reported routine polysomnography for all preterm infants in the context of caffeine discontinuation.

Twenty‐nine per cent of responding centres re‐admit preterm infants for caffeine cessation under inpatient surveillance. Among the 61% of centres which do discontinue caffeine in an outpatient setting, roughly two‐thirds require home monitoring, whereas one third allow caffeine cessation at home without monitoring. The remainder of centres reported more complex standard procedures not captured by the questionnaire.

#### 3.1.1 Influence of Treatment Volume

In high‐volume centres (> 50 VLBW infants per year), a written institutional policy on caffeine use in preterm infants was slightly more common (65% vs. 54%, *p* = 0.27). For most of the other items, no clear difference between high and low volume centres was present, either. One exception was that high‐volume centres relying on a fixed time point after discharge more often used PMA (80% vs. 0%) rather than time since discharge as the discontinuation criterion (*p* = 0.007).

**FIGURE 2:**
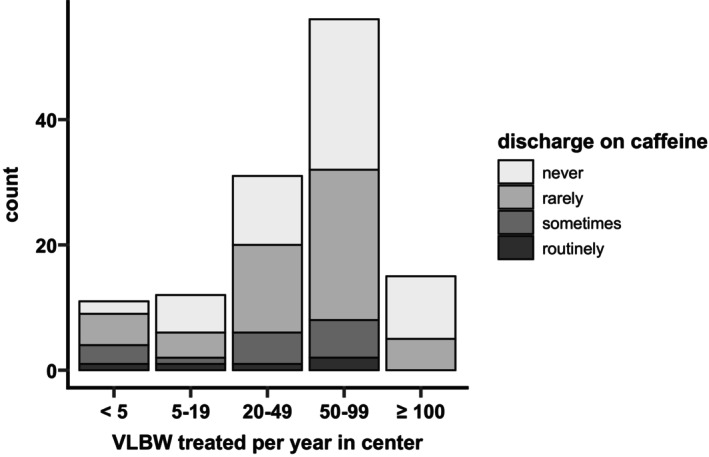
Frequency of centres reporting discharge on caffeine therapy, depending on centre volume. VLBW = very low birthweight infants.

Interestingly, the proportion of centres reporting never discharging preterm infants on caffeine was highest in the highest‐volume group (≥ 100 VLBW/year) (Figure 2), although not statistically significant (*p* = 0.32).

#### 3.1.2 Regional Differences in Management

While centre volume did not predict practises surrounding discharge of VLBW infants on caffeine therapy, we could observe regional differences.

For meaningful comparisons, countries with fewer than four respondents were pooled into regional groups (North: Denmark, Finland, Norway, Sweden; East: Czech Republic, Hungary; Southeast: Albania, Croatia, Romania, Serbia; South: Portugal, Spain; Benelux: Belgium, Netherlands). Countries were taken as entered by the respondents.

**FIGURE 3:**
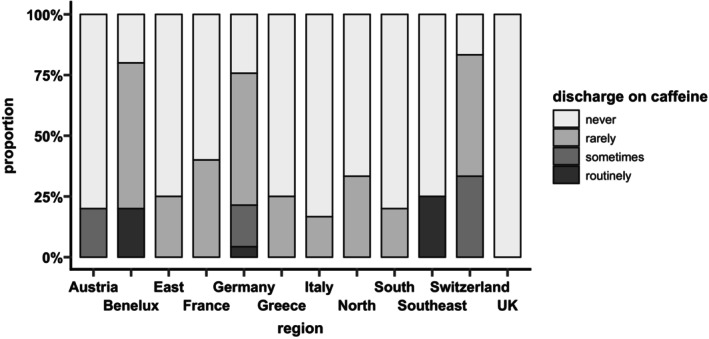
Proportion of centres reporting discharge on caffeine therapy, depending on country/region.

Discharging preterm infants on caffeine therapy appears common in Benelux, Germany and Switzerland, while no respondents from the United Kingdom reported doing so (Figure 3; *p* = 0.01).

**FIGURE 4:**
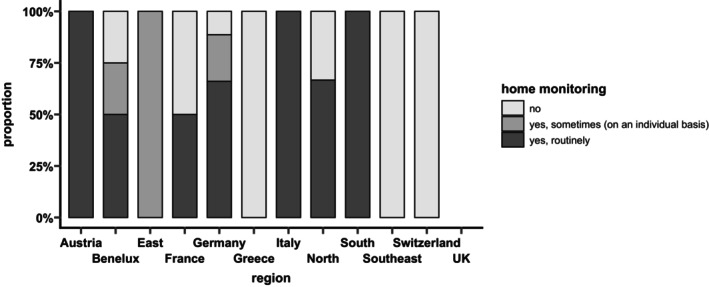
Proportion of centres requiring home monitoring with outpatient caffeine therapy for each country/region.

Policies requiring home monitoring during outpatient caffeine therapy also varied significantly between European regions (*p* = 0.003; Figure 4).

**FIGURE 5:**
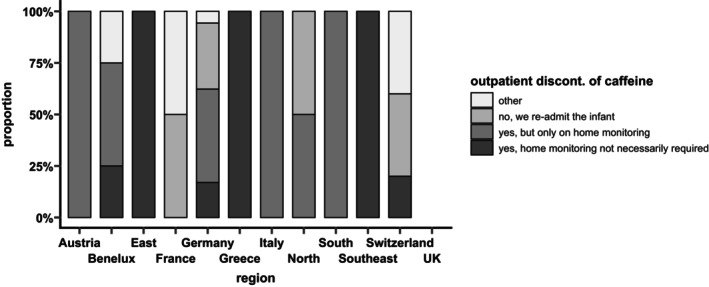
Proportion of centres reporting specific strategies concerning outpatient discontinuation of caffeine therapy for each country/region.

In the context of cessation of outpatient caffeine therapy, again there was considerable variation in practise in regard to readmission or home monitoring (*p* = 0.08; Figure 5).

## 4 Discussion

Since the CAP trial [1], caffeine therapy has become standard of care for premature infants born before 32 weeks PMA. In addition to its immediate effects on apnea of prematurity, there is accumulating evidence on long‐term benefits, in particular on respiratory and neurodevelopmental outcomes [2, 16]. However, despite the widespread use of caffeine in neonatal intensive care, optimal dose and duration of therapy have not been established. Prolonging caffeine therapy beyond 34 weeks PMA appears to reduce intermittent hypoxia in preterm infants [17]. However, the impact on readiness for discharge and the impact on longer‐term outcomes has not been established. Ongoing studies (e.g., NCT06461429, NCT03321734) will hopefully provide guidance in this matter in the future. Possibly, a subgroup of extremely preterm infants with particularly severe respiratory immaturity might benefit from extending caffeine therapy beyond 34 weeks PMA.

We show that extending caffeine therapy beyond discharge of preterm infants is widespread practise across Europe, with considerable regional variation. Some respondents even considered discharge on caffeine as standard therapy, even though robust evidence for this practise is lacking. Of note, the only ready‐to‐use caffeine formulation for preterm infants licenced by the European Medicines Agency is intended solely for in‐hospital use.

Interestingly, no respondent from the United Kingdom reported discharge on caffeine therapy. Although our UK sample was small, this is in line with results from a larger UK‐based sample reporting ambulatory caffeine therapy after NICU care as extremely rare in the UK [18].

Caffeine metabolism and its half‐life are subject to profound changes after preterm birth and in the first year of life. Caffeine half‐life in preterm infants has been reported to decrease from approx. Six to three days from birth to near‐term age [19, 20, 21], while in older children and adults, half‐life appears to be less than 5 h [21]. Re‐occurrence of apnea‐bradycardia events in preterm infants otherwise ready for discharge has indeed been reported up to 7 days after stopping caffeine treatment [19]. Nevertheless, a substantial proportion of respondents stated discharging infants less than 5 days after caffeine cessation routinely.

Likewise, prescription of home monitoring with caffeine therapy was common in some countries, while rare in others.

Practises concerning discontinuation of ambulatory caffeine therapy again varied widely. While some centres stop caffeine therapy without home monitoring and without formal testing, others routinely readmitted the infant and some even required full polysomnography. Systematic data on the safety of ambulatory caffeine therapy and evidence‐based recommendations concerning safe cessation of caffeine therapy are urgently needed, weighing risks and costs as well as family‐important outcomes.

To put this data into context, data from Canadian neonatal units shows similar variation in practise [22]. Discharge on caffeine was not uncommon in this survey, but there was no data on home monitoring and ambulatory discontinuation. In another study comparing academic neonatal centres in the United States, Canada and France, discharge on caffeine therapy was more common in the United States, but still practised frequently in Canada and France. Prescription of home monitoring was very common in the United States while similarly less common in Canada and France [9].

An important limitation of our study is the modest response rate of 20%. Thus, possible sample bias limits the representativeness of our findings. Country‐specific (France, UK) data from our sample seems to be in the previously reported range [9, 18]. However, centres discharging preterm infants on continued caffeine therapy may be over‐represented in our sample. A strength is that we obtained responses from as many as 21 countries, including many lower‐volume centres. This might reflect real‐world practise in the field better than limiting data to academic centres organised in existing networks.

In conclusion, we demonstrate wide variation in practises surrounding discharging preterm infants on continued caffeine therapy across Europe. Interestingly, variation could be attributed to regional differences rather than centre volume or level of care. This seems to reflect the paucity of robust evidence. Given the importance of the subject, evidence‐based guidelines are urgently needed to standardise treatment practises. Our study might help design trials addressing the most pressing questions, grounding possible interventions in the existing heterogeneity of clinical care.

## Author Contributions

**Martin Kuntz:** conceptualization, formal analysis, writing – original draft, writing – review and editing, project administration, investigation, visualization. **Lina Raljevic:** conceptualization, investigation, writing – review and editing, software, project administration. **Katharina Bergmann:** conceptualization, software, project administration, writing – review and editing. **Daniel Matheisl:** conceptualization, writing – review and editing, validation. **Hendryk Schneider:** conceptualization, validation, writing – review and editing. **Hans Fuchs:** conceptualization, writing – review and editing, project administration, resources.

## Funding

The authors have nothing to report.

## Ethics Statement

The survey was prospectively registered with the German Clinical Trials Register (DRKS00036534). It was approved by the Ethics Committee of the University Medical Center Freiburg (application number 24‐1562‐S1).

## Consent

All participants were informed about the study procedures and data privacy prior to providing electronic informed consent.

## Conflicts of Interest

The authors declare no conflicts of interest.

## Supporting information


**Appendix S1:** Codebook export from REDCap.

**Table S1:** Units contacted per country and responses received.

## Data Availability

The data that support the findings of this study are not publicly available due to data privacy concerns but are available from the corresponding author upon reasonable request.
