# Supplementary material for: Discharging Preterm Infants on Caffeine—Practise Variation Across Europe: Results of a Cross‐Sectional Survey
Source: Acta Paediatr. 2026 Mar 14;115(7):1437–43. doi: 10.1111/apa.70502 (PMC13250953; doi:10.1111/apa.70502)
Supplement: Supplementary file 1 — Appendix S1: Codebook export from REDCap. [file APA-115-1437-s002.docx]

**Appendix S1**

Codebook export from REDCap

Instrument: **Discharge on caffeine**

|  | | | |
| --- | --- | --- | --- |
| 77 | [policy1] | Does your center have a written policy concerning the discontinuation of caffeine therapy in premature infants? | radio, Required   \| 1 \| yes \| \| --- \| --- \| \| 2 \| no \| |
| 78 | [when1] | When do you typically discontinue caffeine in a stable infant? | radio, Required   \| 1 \| when off ventilation \| \| --- \| --- \| \| 2 \| when reaching a certain postmenstrual age \| \| 3 \| combination of respiratory stabilty and postmenstrual age \| |
| 79 | [ga1]  Show the field ONLY if:  [when1] = '2' or [when1] = '3' | At which postmenstrual age do you discontinue caffeine in a stable infant? | radio, Required   \| 1 \| < 32 weeks \| \| --- \| --- \| \| 2 \| 32+0 - 33+6 weeks \| \| 3 \| 34+0 - 36+6 weeks \| \| 4 \| ≥ 37+0 weeks \| |
| 80 | [offcaf1] | Do you require infants to be off caffeine for a certain amount of time before discharge? | yesno, Required   \| 1 \| Yes \| \| --- \| --- \| \| 0 \| No \| |
| 81 | [daystodisc1]  Show the field ONLY if:  [offcaf1] = '1' | How long do infants have to be off caffeine before discharge? | slider (number, Min: 0, Max: 21), Required Slider labels: days, ,  Custom alignment: RH |
| 82 | [disc1] | How often do you discharge infants on caffeine? | radio, Required   \| 1 \| routinely \| \| --- \| --- \| \| 2 \| sometimes \| \| 3 \| rarely \| \| 4 \| never \| |
| 83 | [cafdose11]  Show the field ONLY if:  [disc1] = '1' or [disc1] = '2' or [disc1] = '3' | Section Header:  What is your standard dosing of caffeine (free base) for home therapy? | radio, Required   \| 1 \| 5 mg/kg/day \| \| --- \| --- \| \| 2 \| > 5 and < 10 mg/kg/day \| \| 3 \| 10 mg/kg/day \| \| 4 \| > 10 and < 20 mg/kg/day \| \| 5 \| 20 mg/kg/day \| \| 6 \| > 20 mg/kg/day \| \| 7 \| other \| |
| 84 | [cafdosetime1]  Show the field ONLY if:  [disc1] = '1' or [disc1] = '2' or [disc1] = '3' | In how many doses do you administer the prescribed caffeine for home therapy? | radio, Required   \| 1 \| in 1 dose \| \| --- \| --- \| \| 2 \| in 2 doses \| \| 3 \| in >2 doses \| |
| 85 | [doesadj1]  Show the field ONLY if:  [disc1] = '1' or [disc1] = '2' or [disc1] = '3' | Do you adjust the caffeine dose at home with the infant growing? | yesno, Required   \| 1 \| Yes \| \| --- \| --- \| \| 0 \| No \| |
| 86 | [monitor1]  Show the field ONLY if:  [disc1] = '1' or [disc1] = '2' or [disc1] = '3' | Section Header:  Do you prescribe home monitoring when on caffeine? | radio, Required   \| 1 \| yes, routinely \| \| --- \| --- \| \| 2 \| yes, sometimes (on an individual basis) \| \| 3 \| no \| |
| 87 | [regularvisit1]  Show the field ONLY if:  [disc1] = '1' or [disc1] = '2' or [disc1] = '3' | Do you see patients discharged on caffeine on a regular basis in your clinic? | radio, Required   \| 1 \| no \| \| --- \| --- \| \| 2 \| yes, less than monthly \| \| 3 \| yes, monthly \| \| 4 \| yes, every two weeks \| \| 5 \| yes, once a week \| \| 6 \| yes, at least twice a week \| |
| 88 | [disoncttime1]  Show the field ONLY if:  [disc1] = '1' or [disc1] = '2' or [disc1] = '3' | How do you determine when to discontinue caffeine therapy in infants following discharge? | radio, Required   \| 1 \| on an individual basis \| \| --- \| --- \| \| 2 \| fixed timepoint of discontinuation \| |
| 89 | [critdiscon1]  Show the field ONLY if:  [disoncttime1] = '1' | What are your criteria for the discontinuation of caffeine after discharge? Multiple answers are possible. | checkbox, Required   \| 1 \| critdiscon1___1 \| monitor readings \| \| --- \| --- \| --- \| \| 2 \| critdiscon1___2 \| patient history \| \| 3 \| critdiscon1___3 \| current wellbeing \| \| 4 \| critdiscon1___4 \| other \| |
| 90 | [spec1]  Show the field ONLY if:  [critdiscon1(4)] = '1' | In case of "other", please specify. | notes |
| 91 | [timediscon1]  Show the field ONLY if:  [disoncttime1] = '2' | Which fixed timepoint for caffeine discontinuation counts? | radio, Required   \| 1 \| certain timepoint after discharge \| \| --- \| --- \| \| 2 \| certain timepoint after due date \| \| 3 \| whichever of the two timepoints comes first \| |
| 92 | [weekdisc1]  Show the field ONLY if:  [timediscon1] = '1' or [timediscon1] = '3' | What is your fixed timepoint after discharge to discontinue caffeine? Please choose the number of weeks. | slider (number, Min: 0, Max: 16) Slider labels: 0, weeks after discharge, 16 Custom alignment: RH |
| 93 | [weekdisc3]  Show the field ONLY if:  [timediscon1] = '2' or [timediscon1] = '3' | What is your fixed timepoint after due date (40+0 weeks) to discontinue caffeine? Please choose the number of weeks. | slider (number, Min: 0, Max: 16) Slider labels: 0, weeks after due date, 16 Custom alignment: RH |
| 94 | [outpatdisc1]  Show the field ONLY if:  [disc1] = '1' or [disc1] = '2' or [disc1] = '3' | Do you discontinue caffeine in an outpatient setting? | radio, Required   \| 1 \| no, we re-admit the infant \| \| --- \| --- \| \| 2 \| yes, but only on home monitoring \| \| 3 \| yes, home monitoring not necessarily required \| \| 4 \| other \| |
| 95 | [spec2]  Show the field ONLY if:  [outpatdisc1] = '4' | In case of "other", please specify. | notes |
| 96 | [commentend]  Show the field ONLY if:  [disc1] = '1' or [disc1] = '2' or [disc1] = '3' or [disc1] = '4' | Section Header:  Would you like to share any comments on caffeine treatment with us? | notes |
