# Supplementary material for: Discharging Preterm Infants on Caffeine—Practise Variation Across Europe: Results of a Cross‐Sectional Survey
Source: Acta Paediatr. 2026 Mar 14;115(7):1437–43. doi: 10.1111/apa.70502 (PMC13250953; doi:10.1111/apa.70502)
Supplement: Supplementary file 2 — Table S1: Units contacted per country and responses received. [file APA-115-1437-s001.docx]

***Suppl. Tab. S1****: Units contacted per country and responses received.*

| **country** | **sent** | **complete** | **response rate** |
| --- | --- | --- | --- |
| Albania | 1 | 1 | 100% |
| Austria | 7 | 5 | 71% |
| Belgium | 10 | 3 | 30% |
| Bosnia and Herzegovina | 3 | 0 | 0% |
| Bulgaria | 8 | 0 | 0% |
| Croatia | 5 | 1 | 20% |
| Cyprus | 1 | 0 | 0% |
| Czech Republic | 21 | 3 | 14% |
| Denmark | 4 | 2 | 50% |
| Estonia | 2 | 0 | 0% |
| Finland | 5 | 1 | 20% |
| France | 34 | 5 | 15% |
| Germany | 299 | 70 | 23% |
| Greece | 13 | 4 | 31% |
| Hungary | 8 | 1 | 13% |
| Iceland | 1 | 0 | 0% |
| Ireland | 5 | 0 | 0% |
| Italy | 34 | 6 | 18% |
| Latvia | 2 | 0 | 0% |
| Lithuania | 3 | 0 | 0% |
| Luxembourg | 2 | 0 | 0% |
| Moldova | 1 | 0 | 0% |
| Netherlands | 9 | 2 | 22% |
| North Macedonia | 1 | 0 | 0% |
| Norway | 10 | 2 | 20% |
| Poland | 9 | 0 | 0% |
| Portugal | 13 | 2 | 15% |
| Romania | 5 | 1 | 20% |
| Serbia | 4 | 1 | 25% |
| Slovakia | 2 | 0 | 0% |
| Slovenia | 2 | 0 | 0% |
| Spain | 22 | 3 | 14% |
| Sweden | 7 | 1 | 14% |
| Switzerland | 19 | 6 | 32% |
| Turkey | 10 | 0 | 0% |
| United Kingdom | 51 | 5 | 10% |
